# Relying on Visiting Foreign Doctors for Fistula Repair: The Profile of Women Attending Fistula Repair Surgery in Somalia

**DOI:** 10.1155/2017/6069124

**Published:** 2017-07-06

**Authors:** Abdi A. Gele, Abdulwahab M. Salad, Liban H. Jimale, Prabhjot Kour, Berit Austveg, Bernadette Kumar

**Affiliations:** ^1^Institute of Nursing and Health Promotion, Oslo and Akershus University College of Applied Science, Oslo, Norway; ^2^Somali National University, Mogadishu, Somalia; ^3^Daynile Hospital, Mogadishu, Somalia; ^4^Norwegian Center for Minority Health Research, Oslo, Norway

## Abstract

Obstetric fistula is treatable by surgery, although access is usually limited, particularly in the context of conflict. This study examines the profile of women attending fistula repair surgery in three hospitals in Somalia. A cross-sectional study was conducted in Somalia from August to September 2016. Structured questionnaires were administered to 81 women who registered for fistula repair surgery in the Garowe, Daynile, and Kismayo General Hospitals in Somalia. Findings revealed that 70.4% of the study participants reported obstetric labor as the cause of their fistula, and 29.6% reported iatrogenic causes. Regarding the waiting time for the repair surgery, 45% waited for the surgery for over one year, while the rest received the surgery within a year. The study suggests that training for fistula surgery has to be provided for healthcare professionals in Somalia, fistula centers should be established, and access to these facilities has to be guaranteed for all patients who need these services.

## 1. Background

Article 3 of the sustainable development goals requires all nations to improve maternal health by reducing the maternal mortality ratio to 70 per 100,000 births and to achieve universal access to reproductive healthcare services [1]. One of the common causes of maternal mortality is obstructed labor, which is mainly due to a lack of access to professional birth attendants and emergency obstetric services [1]. The consequences of obstetric or prolonged labor can include an obstetric fistula that develops as a result of the prolonged pressure of the presenting fetal part on the pelvis over days of labor, leading to a vesicovaginal fistula, a rectovaginal fistula, or both [2]. Studies on women affected by fistula in Africa found that sociocultural and health system factors contribute to occurrences of obstetric fistula [3]. Early marriage, female genital mutilation, teenage pregnancies, and lack of access to emergency obstetric care are also some of the major risk factors [3]. The most effective way to prevent a fistula is to ensure access to quality maternal healthcare services, including family planning, skilled birth attendance, and emergency obstetric care [4]. Somalia is a country experiencing decades of armed conflict, which not only exposes Somali women to potential risk factors for an obstetric fistula, but also limits their ability to prevent and seek treatment for a fistula. This study examines the profile of women attending fistula repair surgery in three hospitals in Somalia.

An estimated 60,000 to 90,000 women develop an obstetric fistula annually in Sub-Saharan Africa [5]. There are also two million women in Sub-Saharan Africa and Asia who live with a fistula that has not been repaired, thereby enduring the resultant incontinence. However, an obstetric fistula is treatable by surgery, although access is usually limited, particularly in the context of conflict [6]. Fistula repair services in many African countries are overwhelmed by the increased numbers of cases that occur each year [7]. Many countries with a high prevalence of fistula lack adequate health professionals with specialized training to treat fistulas. In Uganda, for example, studies reported a growing number of women with a fistula, a shortage of physicians with the skills to treat fistulas, few and insufficiently equipped operating theatres, and a reliance on visiting doctors to treat the large numbers of women awaiting surgery [7]. A prior meta-analysis reported that more than 110,000 women in Ethiopia presently have a vaginal fistula, but less than 2% of them receive fistula surgery each year [8]. This means it will take at least 55 years to treat the existing patients at the current rate provided no new cases occur [9].

With limited access to fistula surgery, many women in Sub-Saharan Africa continue to live with a fistula and its resultant incontinence for months or years, suffering the devastating physical, psychological, and social consequences of this deeply unpleasant condition. Many of these women are excluded from community life and abandoned by their loved ones, isolating them both socially and economically [10]. Research in Addis Ababa found that women affected by a fistula are rejected by their husbands and they often had to depend on handouts from relatives, with some of the women being reduced to begging [11]. As a result, a significant number of women reported not having the means to afford soap and clothes to stay clean [11]. The most effective short-term strategies for obstetric fistula prevention include an enhanced surveillance of labor, improved access to emergency obstetric services, competent medical care for women, both during and after obstructed labor, and the development of specialist fistula centers to treat injured women, with long-term strategies that include universal access to emergency obstetric care, improved access to family planning services, and increased education for girls and women [12]. Fistulas can be prevented directly during delivery when skilled providers identify women and girls at risk for an obstetric fistula and refer them to fistula prevention centers where they can readily access emergency obstetric care [3]. The failure of nations to provide early fistula treatment and preventive care violates women's internationally recognized human rights, especially in relation to healthcare in general and reproductive healthcare in particular [5].

Somalia is a country that has experienced decades of armed conflict, and it is one of the Sub-Saharan countries where a fistula is common [13]. The indicators of health-related millennium development goals (MDGs) in Somalia are among the worst in the world. The infant mortality rate was estimated at 132 per 1,000 live births, with the under-five mortality rate being 224 per 1,000 live births [14]. Somalia has a maternal mortality of 1000/100,000, making it the third highest maternal mortality rate in the world [15]. Earlier reports show that 58% of districts in Somalia have no professional midwives [7]. While 61% of all childbirths in the world are attended by a skilled birth attendant [5], only 20% of all childbirths in Somalia are attended by trained health personnel, with only 9% of childbirths in rural areas [16]. The achievement of the millennium development goal of improving maternal mortality by reducing three-fourths of the 1990 rate would imply a decrease in the current rate of 1000/100,000 to 250/100,000 by 2015 [1], which Somalia has already failed to achieve given the high prevalence of female genital mutilation (traditional practices that result in modifications of girls' genitals for a reason that is not medical), early marriage, and the limited availability of material and human resources, such as trained staff and emergency obstetric care infrastructure [16]. According to UNFPA data on the midwifery workforce, there are 429 midwives in Somalia, with a density of one midwife per 1,000 live births. Therefore, when complications arise, Somali women either die or develop debilitating conditions, such as obstetric fistulas, and/or lose their babies. This study examines the profiles and experiences of women attending fistula repair surgery in three different regions in Somalia.

## 2. Methods

Fistula surgery has been implemented in three major hospitals in Somalia from August to September 2016 by the NGO called Physician Across Continent (PAC), in collaboration with UNFPA and the Somali Ministry of Health (MOH) [17]. Prior to the initiation of the surgery, an extensive public media campaign was initiated to inform women throughout the country about the upcoming fistula repair program. Women with a fistula were encouraged to seek the surgery through one of three hospitals; Mogadishu (Daynile General Hospital), Kismayo (Kismayo General Hospital), and Garowe (Garowe General Hospital). On August 25 of that year, a project was commenced for 60 women at Garowe General Hospital, who came from different locations from within and outside Puntland. Similarly, 142 women had registered for surgical repairs at Daynile General Hospital. On August 30 of that same year, the team travelled to Kismayo district and conducted clinical check-ups for 24 women who came for fistula repairs [17]. A research team simultaneously informed the management of the hospitals about the study and asked permission to execute it. Women who were not prescribed for repair surgery, as well as those who refused to participate in the research, were excluded from the research. Lastly, structured questionnaires were filled out from nine women who attended the Garowe hospital, 21 women who attended the Kismayo hospital, and 73 women who attended the Daynile hospital, a total of 93 women. However, because questionnaires were not filled correctly from the nine women in the Garowe hospital, we excluded them from the analyses. Moreover, two cases which reported congenital fistulas, as well as a 70-year-old woman who reported having had a fistula for over two decades, were excluded from the analyses, which finally made our sample a total of 81 women. Because the country does not have an ethical committee, we asked permission from the management of the hospitals by presenting the study objectives and procedures for the data collection. We followed common research ethics principles in the carrying out of this study, including informed consent, in which both the right to refuse and withdrawal and confidentiality were explained to each participant. Afterwards, verbal consent was obtained from all the participants.

The questionnaires included sociodemographic details and questions about knowledge of fistulas, female genital cutting, type of marriage, and place of residence for the study participants. In addition, we collected information about the person who attended the labor and perceived barriers to seeking fistula surgery. Moreover, we collected information about the time between the development of the fistula and the first encounter of the fistula repair center. This variable was used as an outcome variable because of its importance in the study context.

A medical doctor and two nurses filled out the questionnaires from participants. The interviewers developed trust and an intimate relationship with the participants prior to initiation of the interviews. Participants were also given information about the study's objectives, as well as the research question that the study intended to answer prior to the interviews. The interviews were conducted in the Somali language, which was the native language of both the participants and the interviewers.

### 2.1. Data Analysis

The Statistical Package for the Social Sciences (SPSS), version 22, was used for the data analysis. Chi-square test was performed for the analyses of categorical variables and a *t*-test for continuous variables. In order to determine the association between exposure and outcome variables, a univariate and multivariate logistic analysis were performed. The association was assessed by using a 95% confidence interval (CI) and an odds ratio (OR), with the level of significance being determined at a value <0.05.

## 3. Results

### 3.1. Characteristics of the Study Participants

Of the study participants, 63% were married, while 37% were either divorced or widowed. The mean age of marriage was 16.8 (SD 2.36). With regard to the type of marriage, 21% were married through a forced marriage, 21% by an arranged marriage, and the rest by choice (Table 1). From an educational prospective, 17.3% had a formal education, while 81.5% had no formal education. Nearly 16% of women were employed, and 81.5% were unemployed. The majority of study subjects, 87.7%, had a vesicovaginal fistula, 8.9% had a rectovaginal fistula, and 3.7% had both.

Majority of the participants (58%) gave birth with the assistant of a traditional birth attendant, whereas only 35.8% gave birth with the assistance of health personnel. As expected, 95% of the participants have experienced female genital mutilation (FGM). 70.4% of the study participants reported obstetric labor as the cause of their fistula, and 29.6% reported iatrogenic causes. As far as the waiting time for the repair surgery, 45% waited for the surgery for over one year, while the rest received the surgery within a year.

Approximately half (49.4%) of the participants resided in areas with a history of intermittent conflict between the government and Islamists; 26% lived in areas controlled by the government, whereas 24.7% resided in areas controlled by Islamists.

Approximately 23.5% of the study participants were divorced because of a fistula, and 11.1% were abandoned by their friends and relatives. One-third (33.3%) reported the lack of money as a barrier to seeking fistula repair surgery; 33.3% did not know any health facility that could perform fistula repair surgery, while 18.5 experienced a combination of both.

A group difference analysis shows that women who were divorced or widowed were more likely to wait for the surgery ≥1 year compared to those who were married (*p* = 0.03). Similarly, women who had no formal education (*p* = 0.002) and those who were unemployed (*p* = 0.01) were more likely to wait for the surgery ≥1 year compared to their counterparts (Table 2). Those who resided in districts that experienced intermittent conflict between the government and Islamists were likely to wait for fistula surgery ≥1 year (*p* = 0.001). Those who were abandoned by their husband and relatives had to wait to seek fistula repair ≥1 year (*p* = 0.004)

As shown in Table 3, the factors that were significantly associated with a long waiting time ≥1 year for fistula repair surgery included being divorced/widowed (OR = 2.75, 95% CI 1.08–6.96), having no formal education (OR = 14.67, 95% CI 1.81–118.73), being unemployed (OR = 5.12, 95% CI 1.28–20.41), living in areas of intermittent conflict (OR = 7.46, 95% CI 1.86–29.8), having experienced more than one surgery (OR = 4.85, 95% CI 1.72–13.71), and a delivery assisted by a traditional birth attendant (OR = 3.25, 95% CI 1.19–8.8).

After controlling women's education (Model 2), women who were divorced were four times more likely to wait for fistula surgery ≥1 year, compared to women who were married (AOR = 3.53, 95% CI 1.23–10.11). Similarly, women who lived in areas that experienced intermittent conflict were over 10 times more likely to wait for repair surgery for ≥1 year, compared to women living in government-controlled areas (AOR = 10.16, 95% CI 2.0–51.43).

After women's education and occupation were controlled for, being divorced continued to be significantly associated with a waiting time for repair surgery for ≥1 year (AOR = 3.68, 95% CI 1.24–10.87). Similarly, women who lived in areas that experienced intermittent conflict between Islamists and the government were over 11 times more likely to wait for corrective surgery ≥1 year (AOR = 11.66, 95% CI, 2.16–62.8).

## 4. Discussion

The study shows that 71% of study participants were married at the age of ≤18, whereas 65.4% become pregnant at the age of 18 or younger. This finding is similar to findings from other Sub-Saharan African countries such as Chad, Guinea, Mali, and Niger, where child marriage is prevalent and half of all teenage women give birth before the age of 18 [18]. Generally speaking, approximately 90% of adolescent births occur in developing countries [19], with 28-29% of women in Sub-Saharan Africa and southern Asia giving birth at the age of 18 [17]. Nonetheless, young girls between the ages of 15–19 are two times more likely to die during pregnancy or childbirth compared to women over age 20 [20]. High levels of teenage marriage and pregnancy are also a commonly reported problem in both conflict and postconflict settings [21]. A study conducted in a refugee camp of Burundian refugees in Tanzania reported a high prevalence of teenage marriage which was associated with low education attainment and a breakdown of culture, poverty, and unstable family relations [22]. A study conducted in Somalia, prior to civil war, reported that marriage before the age of 16 is rare and that the optimum age for the first child is 17–20 among urban societies [23]. Teenage marriage might become more common in Somalia after the onset of civil war, as a study conducted in 1994 showed that approximately 33% of study participants married before the age of 14 years [24]. The high teenage pregnancy rate found in the current study may be due to an absence of schools, lack of jobs, and social activities among adolescents, which makes getting married and having children the sole option for young Somalis. Gender inequities in Somalia may also put girls' lives at greater risk by limiting their opportunities for education, employment, and decisions over their own reproductive health.

With the high prevalence of teenage marriage, type 3 female genital cutting, and high rates of delivery assisted by traditional birth attendants among Somali women [16], it is not unexpected to see a high prevalence of obstetric fistulas given the multiple risk factors that women are exposed to. For instance, 95% of the study participants experienced female genital cutting, with the vast majority experiencing the most severe form, while nearly 60% were assisted by a traditional birth attendant when giving birth. Obstetric fistulas are common in regions where maternal mortality is high because both maternal mortality and obstetric fistulas reflect the absence of functioning emergency obstetric services [4]. This is the case in Somalia, despite a worldwide call to ensure women's rights to acceptable and adequate quality services during labor and delivery [4]. To improve the health of Somali women, it is necessary for the Somali government and international organizations to devote sufficient resources to the abandonment of female genital cutting, teenage pregnancies, improved institutional delivery, and access to emergency obstetric care.

Nearly half of the study participants have waited over one year to get their fistula surgery. The literature reported the economic, social, and psychological consequences of obstetric fistulas, and when the fistula repair is delayed those consequences may become worse and sometimes irreversible [10]. The barriers to fistula surgery reported by the current study include a lack of money for surgery in private hospitals and the absence of fistula surgery in public hospitals in the country. Given the high prevalence of the risk factors for a fistula, it is sad that there are very few health professionals with a competence for fistula surgery in Somalia. According to the world fistula map, there is only one private center in Somalia that performs fistula repair surgery, and there is only one surgeon, in the center, performing the procedure by fee that is too high for most. Because fistula patients are largely poor, they can hardly afford the cost of the surgery. Thus, most fistula care in Somalia is provided by various charities and nongovernmental organizations, which largely “march their services to the sound of their own drums.” Organizations such as the UNFPA often sponsor fistula repair surgery, though their programs are often infrequent and unpredictable with women who receive information and benefits from the project often being the luckiest ones. Women have to wait until international organizations provide funding for a project that hires foreign doctors who perform fistula repair surgery. In the meantime, women with a fistula have to face a challenging life, social deprivation, and isolation, being psychologically stigmatized and depressed, and leading marital and sexual lives that are no longer joyful. The voices of women with fistulas are not yet heard by the Somali government and its partner organizations, which are supposed to be responsible for providing care to them. The 1978 Alma Ata Declaration stated that access to healthcare for all is a human right, and its violation has been described as being unacceptable when the causes for that violation are unjust, avoidable, and unnecessary [25]. We argue that the Ministry of Health and international organizations that work in the health sector in Somalia have underestimated the magnitude of the problem and the importance of fistula prevention and care. Instead of using funds for temporary tasks such as random fistula repair projects, they should look to sustainable solutions such as helping train local professionals on fistula surgery, in addition to funding fistula centers in the country that women can access both physically and economically. Universal access to sexual and reproductive health is essential not only to achieve sustainable development, but also to ensure the needs and aspirations of women in conflict settings, including Somali women, to help realize their health and human rights, which encompass efforts to eliminate preventable maternal morbidity such as fistulas [26].

Factors associated with delayed fistula surgery include place of residence, with women who live in areas with intense conflict between the government and Islamist forces being >11 times more likely to wait for fistula surgery for over one year than those who live in areas that are fully controlled by either the government or Islamists (AOR = 11.66, 95% CI, 2.16–62.8). Areas with intermittent conflict are difficult to access, given the poor infrastructure such as roads, lack of transportation, and fear that people travelling may be targeted by warring factions. Moreover, the areas under armed conflict are very dangerous for health personnel, in addition to a lack of health infrastructure such as hospitals. Similar situation was reported in areas of armed conflict, where medical staff, buildings, and supplies were targeted and destroyed [27]. The International Committee of the Red Cross (ICRC) revealed that violent lethal attacks on patients, healthcare workers, and facilities are widespread in many conflict settings and pose a serious concern to accessing healthcare in such settings [28].

Being divorced is associated with delayed fistula surgery in the current study (AOR 3.68, CI 1.24–10.87). It has also been widely reported that women who experience obstetric fistulas are often abandoned not only by their husbands, but also by their relatives and friends [6, 10, 29]. Consequently, they are left to deal with the huge responsibilities of the family and children alone [6]. The abandonment of women adds to the burden, as an obstetric fistula mostly affects poor women, with many affected women having other complications and being subjected to social discrimination [30].

The important limitation of this study is its cross-sectional design, hence making it difficult to establish the causes. We interviewed women who have had a fistula for different periods of time, with some of them having the condition for a number of years; therefore, the result may be vulnerable to recall bias. Nevertheless, we studied women who have a fistula, which is a topic that has not been previously studied in Somalia; as a result, it presents some highly needed information for the improvement of women's health in Somalia, particularly women affected by fistulas. Historical experience suggests that fistula prevention can only be achieved through the provision of good antenatal screening, delivery assisted by trained health professionals, and prompt access to emergency obstetric services when problems arise [2]. Consequently, training for fistula surgery has to be provided for healthcare professionals in Somalia, fistula centers should be established, and access to these facilities has to be guaranteed for all patients who need these services. The study suggests that only a sustainable political commitment to improving maternal health in general and access to emergency obstetric care in particular are a fundamental condition for the prevention of fistulas in Somalia. Being mindful of this, we advocate initiatives that will generate and sustain an accessible fistula repair surgery for Somali women.

## Figures and Tables

**Table 1:** Characteristics of study participants.

Variables	Frequency	%
	*n* = 81	
*Marital status*		
Married	51	63
Divorced/widowed	30	37
*Education*		
Primary/secondary	14	17.3
No formal education	66	81.5
*Occupation *		
No	66	81.5
Yes	13	16
*Place of residence*		
Government controlled	21	25.9
Intermittent conflict	20	24.7
Controlled by Al-Shabaab	40	49.4
*Age at marriage *		
>18	13	16
≤18	58	71.6
*Type of marriage *		
By choice	46	57
Arranged	17	21
Forced	17	21
*Age of first pregnancy *		
>18	25	30.0
≤18	53	65.4
*Type of fistula *		
Vesicovaginal fistula	71	87.7
Rectovaginal fistula	7	8.6
*The pregnancy in which fistula developed*		
First pregnancy	30	37
Second pregnancy	10	12.3
Third or above	41	51
*Antenatal care at least once*		
Yes	46	56.8
No	35	43.2
*Person who attended the labor *		
Health personnel	29	35.8
Traditional birth attendant	47	58
*Female genital mutilation (FGM) *		
Not cut	4	4.9
Sunna (the mildest form of FGM or type 1)	13	16
Pharaonic (infibulation)	76.5	78.5
*Reported cause of fistula *		
Obstetric complications	57	70.4
Iatrogenic factors	24	29.6
*Number of surgeries *		
One surgery	57	70.4
Two or over	24	29.6
*Number of years having a fistula *		
≤1 yrs	45	55.6
1.1–3 yrs	19	23.5
≥3 yrs	17	21
*Did a fistula affect your ability to work?*		
Yes	54	66.7
No	25	31
*How did having a fistula affect your social life? *		
Did not affect it much	50	61.7
I was divorced	19	23.5
My friends and relatives abandoned me	9	11.1
*Experienced barriers to fistula treatment *		
Lack of money	27	33.3
Lack of health facility that performed fistula surgery	27	33.3
Lack of money combined with no health facility	15	18.5
Others	7	8.6

**Table 2:** Group differences in seeking fistula surgery.

Variables	Waiting for fistula surgery ≤1 year	Waiting for the surgery >1 year	*p* value
	*N* (%)	*N* (%)	
*Marital status*			
Married	33 (64.7)	18 (35.3)	*p* = 0.03
Divorced/widowed	12 (40)	18 (60)	
*Education*			
Primary or higher	13 (93)	1 (7)	*p* = 0.002
No formal education	31 (47)	35 (53)	
*Occupation*			
Employed	3 (23.1)	10 (76.9)	*p* = 0.01
Unemployed	40 (60.6)	26 (39.4)	
*Place of residence*			
Controlled by the government	16 (76)	5 (23.8)	*p* = 0.001
Controlled by Islamists	23 (57.5)	17 (42.5)	
Intermittent conflict	6 (30)	14 (70)	
*Number of surgeries*			
One surgery	38 (66.7)	19 (33.3)	*p* = 0.003
More than one surgery	7 (29.2)	17 (70.8)	
*Did fistula affect your social relations?*			
No	35 (70)	15 (30)	*p* = 0.004
Yes, I was divorced	5 (26.3)	14 (73.7)	
Yes, my friend/relatives abandoned me	4 (44.4)	5 (55.6)	
*The person who attended the labor*			
Health personnel	21 (72.4)	8 (27.6)	*p* = 0.01
Traditional birth attendant	21 (44.7)	26 (55.3)	
*Antenatal care*			
Yes	23 (50)	22 (62.9)	*p* = 0.269
No	23 (50)	13 (37.1)	

**Table 3:** Factors associated with waiting for fistula surgery ≥1 year.

Variables	Model 1 OR (CI)	Model 2 OR (CI)	Model 3 OR (CI)
*Marital status*			
Married	1	1	1
Divorced/widowed	2.75 (1.08–6.96)	3.53 (1.23–10.11)	3.68 (1.24–10.87)
*Education*			
Yes for formal education	1	—	—
No for formal education	14.67 (1.81–118.73)	—	—
*Occupation*			
Yes	1	1	—
No	5.12 (1.28–20.41)	3.73 (0.92–15.11)	—
*Place of residence*			
Government controlled	1	1	1
Islamist controlled	2.36 (0.72–7.72)	1.92 (0.53–6.72)	2 (0.54–7.54)
Intermittent conflict	7.46 (1.86–29.8)	10.16 (2.0–51.43)	11.66 (2.16–62.8)
*Person attending the labor*			
Health personnel	1	1	1
Traditional birth attendant	3.25 (1.19–8.8)	2.33 (0.8–6.7)	2.34 (0.76–7.17)

Model 1 = crude; Model 2 = education adjusted; Model 3 = education and occupation adjusted.
